# Single Primer Enrichment Technology (SPET) for High-Throughput Genotyping in Tomato and Eggplant Germplasm

**DOI:** 10.3389/fpls.2019.01005

**Published:** 2019-08-07

**Authors:** Lorenzo Barchi, Alberto Acquadro, David Alonso, Giuseppe Aprea, Laura Bassolino, Olivia Demurtas, Paola Ferrante, Pietro Gramazio, Paola Mini, Ezio Portis, Davide Scaglione, Laura Toppino, Santiago Vilanova, María José Díez, Giuseppe Leonardo Rotino, Sergio Lanteri, Jaime Prohens, Giovanni Giuliano

**Affiliations:** ^1^DISAFA, University of Turin, Turin, Italy; ^2^COMAV, Universitat Politècnica de Valencia, Valencia, Spain; ^3^ENEA, Italian National Agency for New Technologies, Energy and Sustainable Economic Development, Rome, Italy; ^4^CREA-GB, Research Centre for Genomics and Bioinformatics, Montanaso Lombardo, Italy; ^5^IGA Technology Services Srl, Udine, Italy

**Keywords:** SPET, genotyping, tomato, eggplant, germplasm

## Abstract

Single primer enrichment technology (SPET) is a new, robust, and customizable solution for targeted genotyping. Unlike genotyping by sequencing (GBS), and like DNA chips, SPET is a targeted genotyping technology, relying on the sequencing of a region flanking a primer. Its reliance on single primers, rather than on primer pairs, greatly simplifies panel design, and allows higher levels of multiplexing than PCR-based genotyping. Thanks to the sequencing of the regions surrounding the target SNP, SPET allows the discovery of thousands of closely linked, novel SNPs. In order to assess the potential of SPET for high-throughput genotyping in plants, a panel comprising 5k target SNPs, designed both on coding regions and introns/UTRs, was developed for tomato and eggplant. Genotyping of two panels composed of 400 tomato and 422 eggplant accessions, comprising both domesticated material and wild relatives, generated a total of 12,002 and 30,731 high confidence SNPs, respectively, which comprised both target and novel SNPs in an approximate ratio of 1:1.6, and 1:5.5 in tomato and eggplant, respectively. The vast majority of the markers was transferrable to related species that diverged up to 3.4 million years ago (*Solanum pennellii* for tomato and *S. macrocarpon* for eggplant). Maximum Likelihood phylogenetic trees and PCA outputs obtained from the whole dataset highlighted genetic relationships among accessions and species which were congruent with what was previously reported in literature. Better discrimination among domesticated accessions was achieved by using the target SNPs, while better discrimination among wild species was achieved using the whole SNP dataset. Our results reveal that SPET genotyping is a robust, high-throughput technology for genetic fingerprinting, with a high degree of cross-transferability between crops and their cultivated and wild relatives, and allows identification of duplicates and mislabeled accessions in genebanks.

## Introduction

Single nucleotide polymorphisms (SNPs) are the most abundant type of sequence variation in eukaryotic genomes and have emerged as the most widely used genotyping markers (Mammadov et al., 2012). Genotyping methods rely on different technologies, including next-generation sequencing (Davey et al., 2011), DNA microarrays (Hoheisel, 2006), and polymerase chain reaction (PCR) (Semagn et al., 2014). A widely used method for high-throughput SNP discovery and genotyping is genotyping by sequencing (GBS) based on different reduced−representation sequencing (RRS) approaches, the majority of which are based on the use of restriction enzymes (Elshire et al., 2011; Scheben et al., 2017). One major limitation of GBS is the random distribution of restriction enzyme sites on the genome, and thus the inability to target markers localized within genes, or having a functional significance.

**FIGURE 1:**
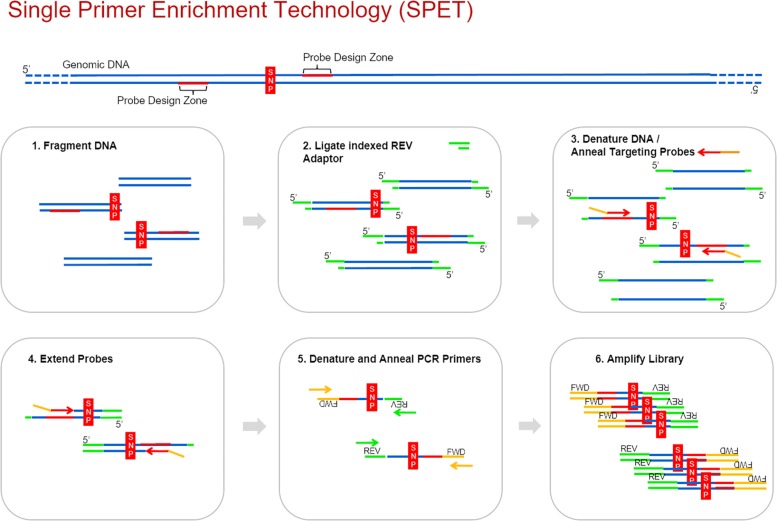
The six main steps of the SPET workflow. Probes can be designed up or downstream the identified SNP.

Recently Nugen^®^ developed the single primer enrichment technology (SPET, United States Patent 9,650,628), which is a customizable solution for targeted sequencing at an affordable price. SPET requires a priori genomic or transcriptomic information and identification of SNPs for probe design. SPET probes are around 40-bases long and are designed adjacent to a region containing a sequence variant, thus enabling detection of both the SNPs and the discovery of additional ones, surrounding the target one (Figure 1). Up to now, SPET has been applied for medical purposes (Scolnick et al., 2015; Nairismägi et al., 2016). Its application to plant materials is still largely unexplored, with one recent exception (Scaglione et al., 2019) assessing SPET application to *Zea mays* L. and to *Populus nigra* L. To date, SPET has not been applied for genotyping of germplasm sets, or of genepools including several related species. The relatively high sequence conservation of exons should facilitate the hybridization of SPET probes designed on these regions across different related species and thus increase the chances to identify novel SNPs, especially if the region downstream of the probe falls in less conserved regions such as introns and Untranslated regions UTR (Castle, 2011). Application of SPET to plant materials for which the genetic diversity and relationships are already known would allow its validation as a reliable and robust high-throughput genotyping method in germplasm sets.

Tomato (*Solanum lycopersicum* L.) and eggplant (*S. melongena* L.) are amongst the economically most important vegetables, and the diversity and genetic relationships of their genepools has been extensively studied. Several studies have applied sequence-, PCR- or microarray-based genotyping in tomato and eggplant, to analyze the genetic diversity and population structure of a limited number of cultivars, breeding lines, landraces or cultivated, and wild relatives (e.g., Vilanova et al., 2012; Cericola et al., 2013; Acquadro et al., 2017; Pailles et al., 2017; Tranchida-Lombardo et al., 2018).

Cultivated tomato materials maintained in germplasm collections include traditional varieties and heirlooms which, compared to its wild relatives, display a narrow genetic diversity resulting from several bottlenecks during domestication and spread (Blanca et al., 2015). Regarding wild species, many studies have been performed evaluating the relationships between them and cultivated tomato (Rodriguez et al., 2009; Aflitos et al., 2014; Lin et al., 2014; Dodsworth et al., 2016; Beddows et al., 2017). The general consensus, using different molecular approaches, is that within the core tomato clade (*Solanum* section *Lycopersicon*), the wild species genetically closest to cultivated tomato are those of the “Lycopersicon” group (Peralta et al., 2008), including *S. pimpinellifolium* L. and the Galápagos Islands endemisms S. *cheesmaniae* (L. Riley) Fosberg and *S. galapagense* S.C. Darwin & Peralta. While the genetic diversity of the latter two species is limited (Pailles et al., 2017), *S. pimpinellifolium* is much more diverse than cultivated tomato heirlooms (Caicedo and Schaal, 2004; Razali et al., 2018). The next closest wild species to the “Lycopersicon” group are those of the “Arcanum” group, which includes *S. arcanum* Peralta, *S. chmielewskii* (C.M. Rick, Kesicki, Fobes & M. Holle) D.M. Spooner, G.J. Anderson & R.K. Jansen, and *S. neorickii* D.M. Spooner, G.J. Anderson & R.K. Jansen, followed by the five species of the “Eriopersicon” group (*S. huaylasense* Peralta, *S. chilense* (Dunal) Reiche, *S. corneliomulleri* J.F. Macbr, *S. peruvianum* L. and *S. habrochaites* S. Knapp & D.M. Spooner), and *S. pennellii* Correll, the only species included in the monotypic “Neolycopersicon” group (Rodriguez et al., 2009; Aflitos et al., 2014; Lin et al., 2014; Dodsworth et al., 2016; Beddows et al., 2017).

Unlike tomato, eggplant belongs to the *Solanum* subgenus *Leptostemonum*, collectively known as the “spiny solanums” group (Vorontsova et al., 2013). Several species from the eggplant clade, such as the direct wild ancestor *S. insanum* L. and several close relatives such as *S. incanum* L., *S. lichtensteinii* Willd., and *S. linnaeanum* Hepper & P-M.L. Jaeger are closely related to eggplant (Vorontsova et al., 2013; Acquadro et al., 2017). Other more distant species include some from the Anguivi grade, which comprises the two other cultivated species: scarlet eggplant (*S. aethiopicum* L.) and gboma eggplant (*S. macrocarpon* L.) and their respective wild ancestors *S. anguivi* Lam. and *S. dasyphyllum* Schumach. & Thonn. as well as other species of potential for breeding such as *S. tomentosum* L. (Kouassi et al., 2016; Plazas et al., 2016). A much more distant group includes American species (Vorontsova et al., 2013; Syfert et al., 2016; Acquadro et al., 2017), among which *S. torvum* Sw. and *S. sisymbriifolium* Lam. represent a potential source of tolerance to diseases for eggplant breeding (Daunay and Hazra, 2012).

Here, we report the application of SPET genotyping, assessing its reliability and using both target and non-target SNPs for studying the genetic variation and population structure of a large set of accessions from cultivated and wild genepools of tomato and eggplant, and to validate them against previous results on their diversity and genetic relationships. The results obtained highlight the potential of the SPET technology for genotyping and management of germplasm collections.

## Materials and Methods

### Single Primer Enrichment Technology (SPET) Set Up

Tomato SNP data were retrieved from the SOL Genomics portal^1^ and specifically from the “150 Tomato Genome Resequencing Project” (Aflitos et al., 2014) and the “AGIS Tomato 360 Resequencing Project” (Lin et al., 2014). Respectively, 52 samples from “150 Tomato Resequencing Project” and 184 samples from “AGIS Tomato 360 Resequencing Project” were used to mine alleles of *S. lycopersicum*. To identify inter-varietal alleles across *S. lycopersicum*, *S. pimpinellifolium*, and *S. lycopersicum* var. *cerasiforme*, 159 additional samples were used from the “AGIS Tomato 360 Resequencing Project”. All resequenced accessions used in this work are available through the SOL Genomics FTP site^2^ and listed in Supplementary Table S1.

Accessions from different VCF files were merged, retaining only simple biallelic SNPs. Since SNP calling was based on ITAG SL2.50 genome build, this was maintained as reference through the analysis with respective gene models. SNP selection was then made based on the following criteria: (i) only positions with alternative cohort-wise allele count greater than 8 (summing two from homozygous loci or one from heterozygous loci), (ii) SNPs within introns and UTRs had to be at least 15 kbp apart from each other or with SNPs in CDS, (iii) SNPs within CDS had to be at least 5 kbp apart from other selected SNPs, and (iv) SNPs have to reside on anchored chromosomes.

For the eggplant panel design sequencing data provided by the Universitat Politècnica de València and by the Italian Eggplant Consortium, which included the whole-genome resequencing of eight *S. melongena* and one *S. incanum* accessions (Gramazio et al., submitted), were aligned against the “67/3” eggplant reference genome (Barchi et al., 2019) with BWA-MEM aligner with default parameters (Li, 2013), discarding multiple-mapping reads. Samtools was used for variant calling (Li et al., 2009). Homozygous/heterozygous SNP calls were considered only with phred-scaled genotype likelihood equal to zero. Similarly to tomato, only biallelic SNPs with an alternative allele count of 4 and a minor allele frequency greater than 0.25 were retained.

From the eligible polymorphic sites previously identified in both the species, a randomly selected panel of SNPs were forwarded for probe design to NuGen (San Carlos, CA, United States). These filtered tomato and eggplant panels were then tested for their sequencing performances and reproducibility, and to identify a final set of about 5k probes for genotyping via SPET, commercialized under the name of Allegro^®^. To this purpose, 24 accessions of both *S. lycopersicum* and *S. melongena* were used in a pilot experiment. In eggplant, the re-call performance was assessed with filtered VCF files using as parameters: (i) homozygous states called with a minimum of 10 reads; (ii) MAF (minor allele frequency) >0.04; (iii) heterozygosity comprised between MAF 0.25–0.5; (iv) a minimum genotyping ratio of 80% (i.e., 80% of samples must satisfy the above constraints). This allowed to retain 7,662 out of 11,625 SNPs (72%).

Besides cross-species genomic sequence conservation and the frequency of expected SNP detection, in both crops, probes were first filtered based on their ability to hit the target SNPs in the first 25 bp after 3′-end of the probe. After the first pilot run with about twice the number of probes, a coverage analysis was used to select the final set of 5,000 and 5,082 probes in tomato and eggplant respectively. Sites showing an average coverage ranging from 46× to 90× and with less than four samples having five or less mapped reads were retained for tomato, while sites with a coverage range of 79–130× and excluding all those probes with three or more individual showing a coverage below 5× were selected for eggplant. Furthermore, 82 extra probes were added to the final eggplant set for specific functional purposes. Sequencing yields, coverage analysis, and filtering for probe selection are provided in Supplementary File S1.

### Plant Material

For tomato, a set of 400 G2P-SOL project^3^ accessions maintained at Universitat Politècnica de València (Valencia, Spain) were included in the study. They comprise 361 accessions of *S. lycopersicum*, 20 of *S. pimpinellifolium*, the closest wild ancestor of the cultivated tomato (Blanca et al., 2015), which has repeatedly served as a source of valuable traits for its improvement (Caicedo and Schaal, 2004), and 19 accessions of six other wild relatives. The latter include three species belonging to the “Arcanum” group (i.e., *S. arcanum*, *S. chmielewskii*, and *S. neorickii*), two to the “Eriopersicon” group (i.e., S. *huaylasense* and *S. habrochaites*) and *S. pennellii* belonging to the monotypic “Neolycopersicon” group (Supplementary Table S2). Two DNA samples of the tomato inbred line “Heinz 1706” (CTR_H1_122 and CTR_H1_123) were used as controls.

For eggplant, a set of 422 accessions from the G2P-SOL project were included in the study. According to passport data, the accessions, maintained at Universitat Politècnica de València (Spain) and at the CREA-GB (Montanaso Lombardo, Italy), comprise 362 accessions of *S. melongena* of the Occidental and Oriental groups (Vilanova et al., 2012), 36 and 9 accessions, respectively, of the cultivated *S. aethiopicum* and *S. macrocarpon*, as well as 15 accessions belonging to seven wild relatives (Supplementary Table S3). The latter include four species from the Old World, of which two (*S. incanum* and *S. linnaeanum*), together with *S. melongena*, are part of the “Eggplant” clade, and two other (*S. anguivi* L. and *S. tomentosum* L.), which together with cultivated *S. aethiopicum* and *S. macrocarpon*, are part of the “Anguivi” grade, while three are from American origin (*S. paniculatum* L., *S. sisymbriifolium*, and *S. torvum*) (Miz et al., 2008; Syfert et al., 2016). As control, three DNA samples of the eggplant inbred line “67/3” (i.e., GPE001970, GPE001970b, and control 31) were also genotyped.

### DNA Extraction, Library Construction Preparation, and Sequencing

DNA was extracted using the Qiagen plant mini-prep, the LGC Sbeadex kit or by a modified CTAB method. Libraries were prepared according to the Ovation Rapid Library Systems (Nugen) specifications. The streamlined workflow consists of six main steps, as shown in Figure 1.

For the pilot test, sequencing was performed with the Illumina HiSeq 2500 platform (Illumina Inc., San Diego, CA, United States), following the manufacturer protocol and using 75SE chemistry. For the genotyping of the whole set of accessions with the custom 5K probe sets, sequencing was performed with Illumina HiSeq 2500 platform (Illumina Inc., San Diego, CA, United States), following the manufacturer protocol and using 150SE chemistry. The sequencing raw data are available at NCBI SRA (BioProject ID PRJNA542237 for tomato data and BioProject ID: PRJNA542231 for eggplant data).

### SPET Sequencing and SNP Calling

Base calling and demultiplexing were carried out using the standard Illumina pipeline. The read quality check and adapter trimming was carried out using ERNE (Del Fabbro et al., 2013) and Cutadapt (Martin, 2011) software. After alignment to the reference eggplant and tomato genomes, using BWA-MEM (Li, 2013) with default parameters, the uniquely aligned reads were selected (i.e., reads with a mapping quality >10). SNP calling was obtained with GATK 4.0 (DePristo et al., 2011), following the software best practices in June 2018 for germline short variant discovery^4^. Main steps of the analysis were: (i) per-sample variants calling on target regions using HaplotypeCaller (Poplin et al., 2017), resulting in GVCFs file for each sample; (ii) GVCFs consolidation across multiple samples, in order to improve scalability and speed up the following step using ImportGenomicsDB; (iii) joint genotyping based on GenotypeGVCFs to produce a set of joint-called variants; and (iv) selection of SNPs (using SelectVariants) and quality filtering of SNPs using VariantFiltration (filter expression used: QD < 2.0 || MQ < 40.0 || MQRankSum <−12.5).

To extract high confidence SNPs, Vcftools (Danecek et al., 2011) was applied to both the eggplant and tomato generated VCFs, using the following parameters: min-meanDP 30, max-missing 0.95 (0.80 for tomato) and non-ref-ac-any 1.

### Genetic Relationships Analysis

The polymorphic information content (PIC) of each SNP was evaluated by applying the following equation, as suggested by Anderson et al. (1993): PIC = 1-Σ Pij^2^, where Pij represented the frequency of the jth allele at the ith SNP and the summation was extended over n alleles.

Genetic relationships were described by constructing a phylogenetic tree by maximum likelihood (ML) method using the IQ-TREE software (Nguyen et al., 2015); data are available in Supplementary File S2. Branch supports were obtained with the ultrafast bootstrap (Hoang et al., 2018). Comparison between ML trees was assessed using the Robinson-Foulds distance (Robinson and Foulds, 1981) calculated with ETE 3 (Huerta-Cepas et al., 2016). A principal component analysis (PCA) was obtained with SNPrelate (Zheng et al., 2012) program. Analyses were performed using target SNPs only or using all (target plus non-target) SNPs.

## Results

### SPET Assay Design and Robustness

In tomato, 344,373 eligible SNPs were identified within *S. lycopersicum* (including *S. lycopersicum* var. *cerasiforme*) and *S. pimpinellifolium* using the data from the “150 Tomato Genome Resequencing Project” and the “AGIS Tomato 360 Resequencing Project”, of which 14,566 sites were selected for probe design. Of these, 40% were localized in coding regions and the rest in introns or UTRs. In eggplant, 72,739 eligible SNPs were identified, using a resequencing panel including eight *S. melongena* and one *S. incanum* accessions (Gramazio et al., submitted), of which 11,928 were selected for probe design, being 60% localized in coding regions and the rest in introns or UTRs.

A pilot study was run on 24 genotypes for each species using 75-bases long sequencing, of which 40 bp corresponded to the probe sequence. After sequencing, 13,615 (93%) and 11,265 (94%) probes were found to target SNPs within the first 25 bp after the probe 3′ end in tomato and eggplant, respectively.

**FIGURE 2:**
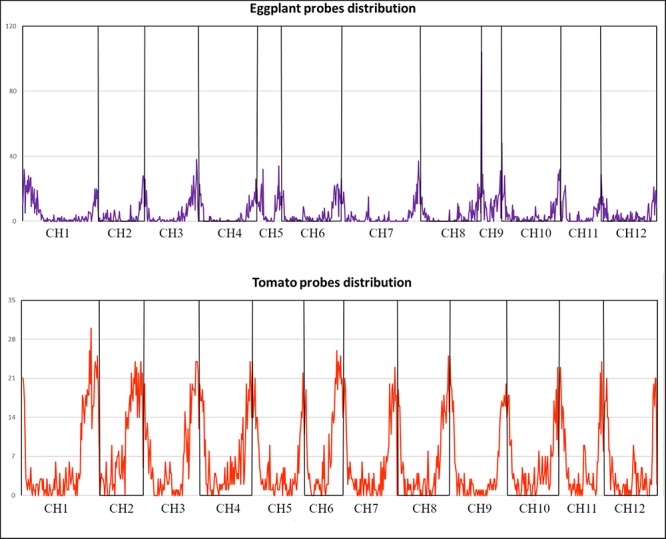
Eggplant and tomato distribution of the 5k SNPs panels according to their genomic position.

Based on sequencing coverage and missing data (see section “Materials and Methods”), a final set of 5,000 probes for tomato (of which 2,254 in CDSs and 2,746 in Introns/UTRs), and 5,082 for eggplant (of which 3,619 in CDSs and 1,463 in Introns/UTRs) were chosen (Supplementary File S1). The probes localized to the gene-rich chromosome arms in both species (Figure 2), and their sequence is reported in Supplementary File S3.

To assess the robustness and transferability of the SPET method, the numbers of reads obtained with the final probe panels and their mapping efficiency were assessed using different DNA preparations (Supplementary Table S4). As can be seen, both the number of reads and the mapping percentage were relatively stable when DNAs prepared with different DNA extraction methods and by different laboratories were used.

### SPET Diversity Assessment of the Tomato Germplasm Set

Using the final panel, around 215 million 150-bp single reads were produced in tomato, yielding 110 bp of useful sequence after probe trimming. After quality filtering, 198 high-quality million reads were retained (8% discarded) for the alignment to the “Heinz 1706” tomato genome sequence version 2.5 (The Tomato Genome Consortium, 2012) with an average mapping rate of 92.2%. Three out of 400 accessions gave an average read depth <10. By applying stringent criteria, 12,002 SNPs were identified among the 397 accessions included in the study. Of these, 4,577 SNPs were those originally targeted by the 5k probes set, while the remaining 7,425 were accessory non-target SNPs. By using the whole set of identified SNPs, the PIC ranged from 0.002 to 0.539 with an average of 0.094 (Supplementary Table S5), while by considering the target SNP panel, and the PIC average raised to 0.147 (Supplementary Table S6).

The 358 *S. lycopersicum* accessions with a read depth >10 showed very low levels of missing data (1.2%), high identity with the Heinz 1706 reference sequence (96.5%) and a low level of heterozygosity (0.65%), compatible with the autogamous reproduction of cultivated tomato (Supplementary Table S2). The missing data were slightly higher for the wild species, due to the sequence polymorphisms underlying the probes, ranging from 1.7% in *S. pimpinellifolium* to 4.2% in *S. neorickii*. Only *S. habrochaites* displayed high missing data (average of 10.4%). Conversely, the identity with the Heinz 1706 reference sequence was lower in wild species, ranging on average from 66.0% in *S. pimpinellifolium* to 43.2% in *S. huaylasense*. The heterozygosity level of wild species was higher than *S. lycopersicum*, ranging from 4.6% in *S. pimpinellifolium* to 27.5% in *S. pennellii* on average, consistent with the partial or total allogamy reported for these species (Chen and Tanksley, 2004). As expected, in the two replicates of the inbred line “Heinz 1706,” more than 99.8% of SNPs showed the same allele of the reference sequence and just 18 and 19 sites, respectively, had alternative/heterozygous SNPs.

### SPET Diversity Assessment of the Eggplant Germplasm Set

In eggplant, more than 252 million single reads were produced. Sequences were trimmed and quality filtered to 242 million useful reads (4% discarded), corresponding to about 600K reads per sample on average. The latter were then aligned to the recently produced reference “67/3” eggplant genome (Barchi et al., 2019) with an average mapping rate of 95.8%. Three out of 422 accessions gave an average read depth <10. By applying stringent criteria, a total of 30,731 polymorphic sites were identified among the 422 accessions included in the study. Among them, 4,628 were SNPs targeted by the 5k probes set, while the remaining 26,103 were accessory non-target SNPs. By using the whole set of identified SNPs the PIC ranged from 0.002 to 0.607 with an average of 0.105 (Supplementary Table S7), while by considering the target SNP panel, the PIC ranged from 0.002 to 0.607 with an average of 0.381 (Supplementary Table S8).

The 360  *S. melongena* accessions with a read depth >10 showed, on average, extremely low levels of missing data (0.02%), high identity with the “67/3” reference sequence (93.6%) and a low level of heterozygosity (0.67%), compatible with the autogamous reproduction of cultivated eggplant (Supplementary Table S3). As for tomato, the missing data were slightly higher for the wild species, ranging from 0.5% in *S. incanum* to 4.5% in *S. macrocarpon*. Only the distantly related species *S. torvum* and *S. sisymbriifolium* displayed high missing data (21.2 and 22.7%, respectively). The identity with the “67/3” reference genome was lower in the eggplant relatives, ranging from 85.9% in *S. incanum* to 50.6% in *S. sisymbriifolium*. The heterozygosity level of wild species and cultivated relatives *S. aethiopicum* and *S. macrocarpon* was higher than that of *S. melongena*, ranging from 1.7% in *S. tomentosum* and *S. aethiopicum* to 9.4% in *S. sisymbriifolium*, consistent with the partial allogamy reported or suggested for these species (Daunay et al., 2001; Vorontsova and Knapp, 2016; Acquadro et al., 2017). As expected, in the three replicates of the eggplant inbred line “67/3” used as a control, over 99.9% of SNPs showed the same allele of the reference genome, and just from 26 to 35 sites showed alternative/heterozygous SNPs.

### Genetic Relationships in the Tomato and Eggplant Germplasm Sets

**FIGURE 3:**
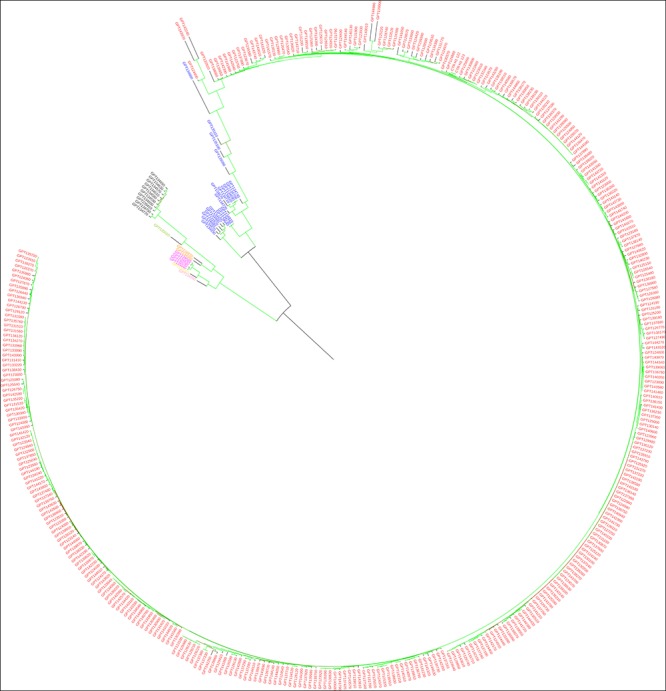
Maximum likelihood phylogenetic tree obtained with IQ-TREE, based on the whole set of SNPs, illustrating the genetic architecture of tomato and wild related species accessions in study. *Solanum lycopersicum* entries are in red, *S. pimpinellifolium* in blue, *S. chmielewskii* in yellow, *S. neorickii* in purple, *S. arcanum* in pink, *S. pennellii* in green, *S. habrochaites* in dark and *S. huaylasense* in orange. Branches are colored according to the bootstrap values (red = 15 and green = 100).

**FIGURE 4:**
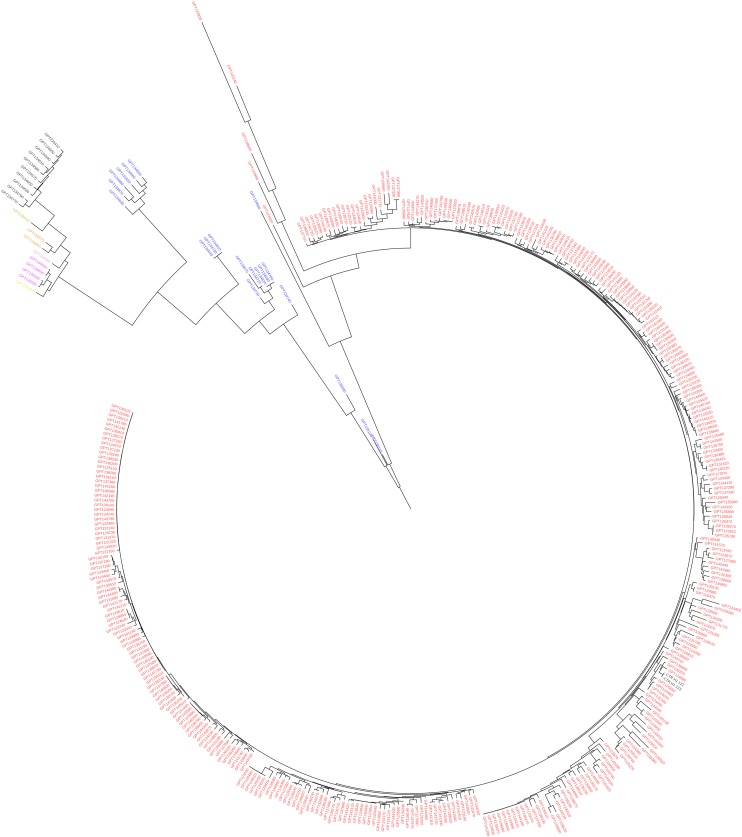
Maximum likelihood phylogenetic tree obtained with IQ-TREE, based on the target SNPs, illustrating the genetic architecture of tomato and wild related species accessions in study. *Solanum lycopersicum* entries are in red, *S. pimpinellifolium* in blue, *S. chmielewskii* in yellow, *S. neorickii* in purple, *S. arcanum* in pink, *S. pennellii* in green, *S. habrochaites* in dark, and *S. huaylasense* in orange. Branches are colored according to the bootstrap values (red = 15 and green = 100).

The maximum likelihood (ML) dendrograms (Figures 3, 4) show the genetic relationships between the tomato accessions in the study. As expected, the replicated samples of the same reference genotypes (CTR_H1_122 and CTR_H1_123) clustered together. In the dendrogram obtained using the whole set of SNPs (Figure 3), the accessions of the “Eulycopersicon” group (i.e., *S. lycopersicum* and *S. pimpinellifolium*) cluster together in a main branch, in which *S. pimpinellifolium* accessions are basal to the monotypic *S. lycopersicum* cluster. The *S. pimpinellifolium* accessions do not intermingle with the ones of *S. lycopersicum*, and they are divided in two branches, one which contains only Peruvian accessions, and another one basal to all *S. lycopersicum* that contains all accessions from Ecuador and three accessions from Peru. In the dendrogram, a second branch includes the other wild species and two main sub-clusters, of which one contains the three “Arcanum” group accessions (*S. arcanum*, *S. chmielewskii*, and *S. neorickii*), with *S. arcanum* basal to the two other species, and the other accessions of *S. huaylasense* and *S. habrochaites* from the “Eriopersicon” group, and *S. pennelli* from the “Neolycopersicon” group, with *S. huaylasense* basal to the two other species.

Some of the main findings obtained using the whole set of SNPs were confirmed using target SNPs (Figure 4), as in both cases the different species are not intermingled. Notwithstanding, the two dendrograms present some differences in terms of topology and branch length, confirmed by the Normalized Robinson-Foulds distance being 0.63. In the dendrogram based on target SNPs, *S. pimpinellifolium* is basal to the rest of accessions which are split in the two major clusters, one containing the *S. lycopersicum* accessions and one accession of *S. pimpinellifolium*, and the other containing the rest of wild species. In this latter, the major branch *S. pimpinellifolium* is spread in different branches which are basal to the rest of wild species, which largely display the same topology than in the dendrogram obtained with all the SNPs (Figure 3).

**FIGURE 5:**
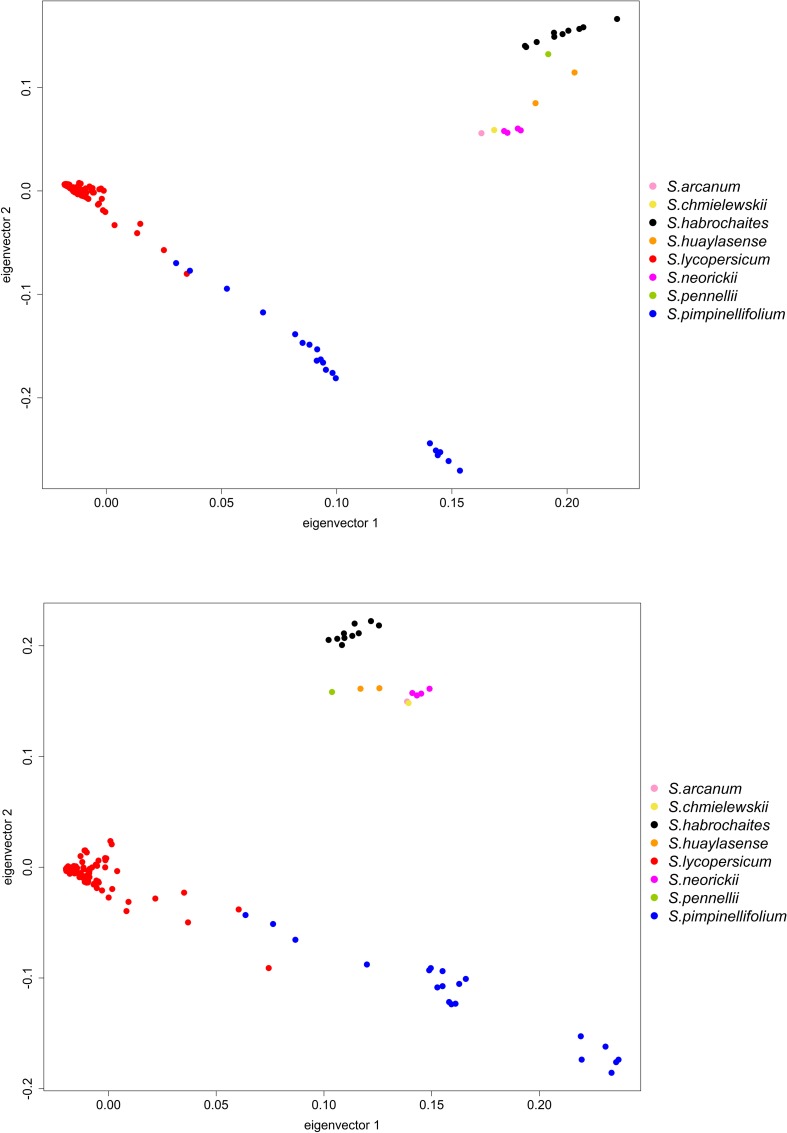
PCA visualization of the genetic relationships among the accessions of tomato and wild related species in study, based on the whole (upper figure) or target (lower figure) SNP Datasets.

PCA analysis for the tomato set, based on the whole set of SNPs (Figure 5) confirmed the grouping of the ML dendrogram. The first and second components accounted for 21.7 and 13.6% of the genetic variation. *S. lycopersicum* and *S. pimpinellifolium* were clearly separated from the other species and all in all well differentiated with a minor degree of overlap. The other wild species are not intermingled and cluster in a different area of the PCA plot (Figure 5). The three species of the “Arcanum” group (*S. arcanum*, *S. chmielewskii*, and *S. neorickii*) are the closest to the “Eulycopersicon” (*S. lycopersicum* and *S. pimpinellifolium*), the species *S. habrochaites* (“Eriopersicon”), and *S. pennellii* (“Neolycopersicon”) are the most genetically distant, while *S. huaylasense* accessions occupy an intermediate position (Figure 5). The first and second axes of the PCA using only target SNPs account for 38.3 and 7.1%, of the genetic variation, respectively (Figure 5), and largely confirm the results of the PCA based on all SNPs. However, *S. lycopersicum* and *S. pimpinellifolium* display a greater dispersion as highlighted in Figure 5.

**FIGURE 6:**
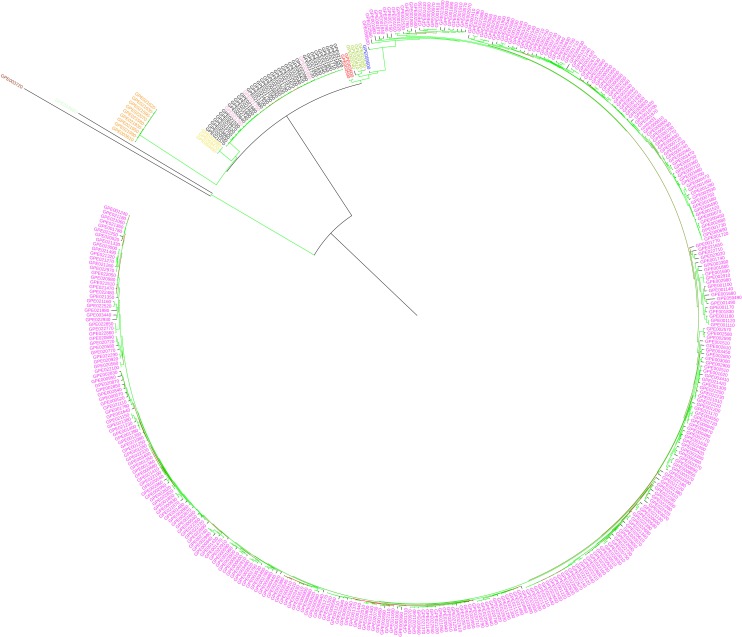
Maximum likelihood phylogenetic tree obtained with IQ-TREE, based on the whole set of SNPs, illustrating the genetic architecture of the accessions of eggplant as well as cultivated and wild related species in study. *Solanum melongena* entries are in purple, *S. aethiopicum* in black, *S. macrocarpon* in orange, *S. anguivi* in pink, *S. incanum* in blue, *S. linnaeanum* in dark green, *S. paniculatum* (mislabeled in the germplasm collection; actually *Solanum* sp.) in red, *S. sisymbriifolium* in brown, *S. tomentosum* in yellow, and *S. torvum* in light green. Branches are colored according to the bootstrap values (red = 25 and green = 100).

**FIGURE 7:**
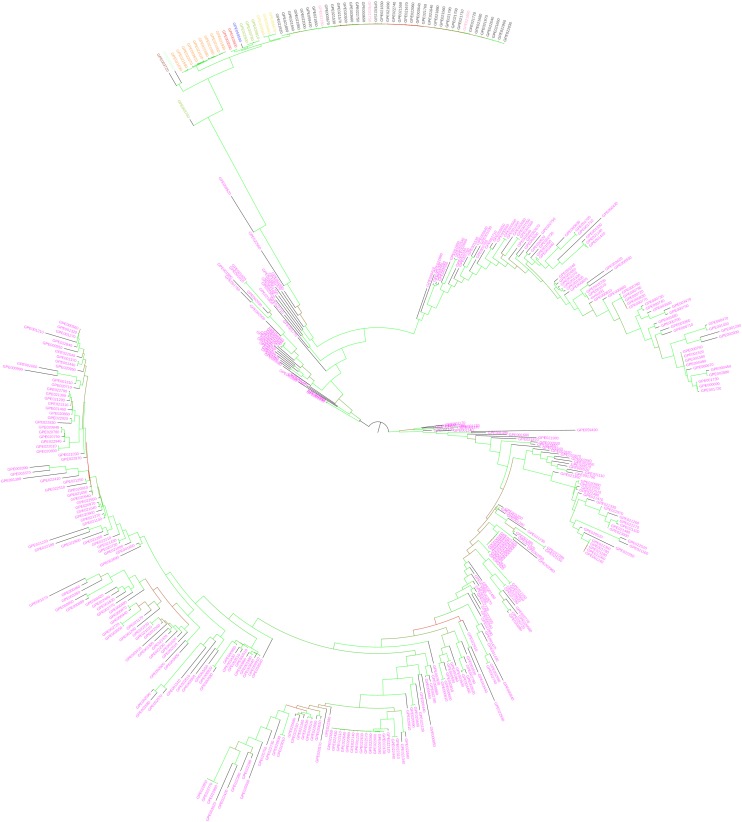
Maximum likelihood phylogenetic tree obtained with IQ-TREE, based on the target SNPs, illustrating the genetic architecture of the accessions of eggplant as well as cultivated and wild related species in study. *Solanum melongena* entries are in purple, *S. aethiopicum* in black, *S. macrocarpon* in orange, *S. anguivi* in pink, *S. incanum* in blue, *S. linnaeanum* in dark green, *S. paniculatum* (mislabeled in the germplasm collection; actually *Solanum* sp.) in red, *S. sisymbriifolium* in brown, *S. tomentosum* in yellow, and *S. torvum* in light green. Branches are colored according to the bootstrap values (red = 29 and green = 100).

In eggplant, the ML-based dendrograms (Figures 6, 7) display the genetic relationships between the accessions in study. As expected, the three replicated samples of the reference genotype cluster together. The dendrogram obtained using the whole SNPs panel identifies two main branches, of which one includes the two accessions from American species (*S. sisymbriifolium* and *S. torvum*), and the other includes the rest of species native to the Old World (Figure 6) together with the two accessions labeled as *S. paniculatum*. In this latter cluster, two major branches are distinguishable: one containing the four species of the “Anguivi” grade (*S. aethiopicum*, *S. anguiv*i, *S. macrocarpon*, and *S. tomentosum*), and one including the three species of the “Eggplant” clade (*S. melongena*, *S. incanum* and *S. linnaeanum*), plus the two *S. paniculatum* accessions. In the cluster of the “Anguivi” grade, the four species are separated in different sub-branches, except *S*. *aethiopicum* and *S*. *anguivi*, which are intermingled. In the other cluster, a branch contains the “Eggplant” clade cluster, in which all the accessions of *S. linnaeanum* are separated in a sub-branch and *S. melongena* and *S. incanum* accessions in another sub-branch, with the latter basal to the monotypic eggplant cluster. The two accessions labeled as *S. paniculatum* are basal to the “Eggplant” clade branch. Three *S. melongena* accessions initially mis-labeled as *S. aethiopicum* in the germplasm bank list clustered correctly with the rest of *S. melongena* accessions.

The eggplant dendrogram based on target SNPs (Figure 7) confirms a good separation among species and the intermingling in the same cluster of *S. aethiopicum* and *S. anguivi*. Discrepancies, confirmed by the Normalized Robinson-Foulds distance equal to 0.53, were observed in the topology and branch lengths compared to the dendrogram obtained with the whole SNPs dataset. The dendrogram based on target SNPs identifies two main branches, one of which includes only *S. melongena* accessions, while the other contains the rest of *S. melongena* accessions, and in a sub-cluster the other cultivated and wild species. In this latter, the two American species *S. sisymbriifolium* and *S. torvum* cluster together and are basal to all the others (Figure 7), while, unexpectedly, the *S. linnaeanum* accession GPE003740 (for which a low number of reads was obtained) shows an odd position, and it appears genetically differentiated (basal) from the others.

**FIGURE 8:**
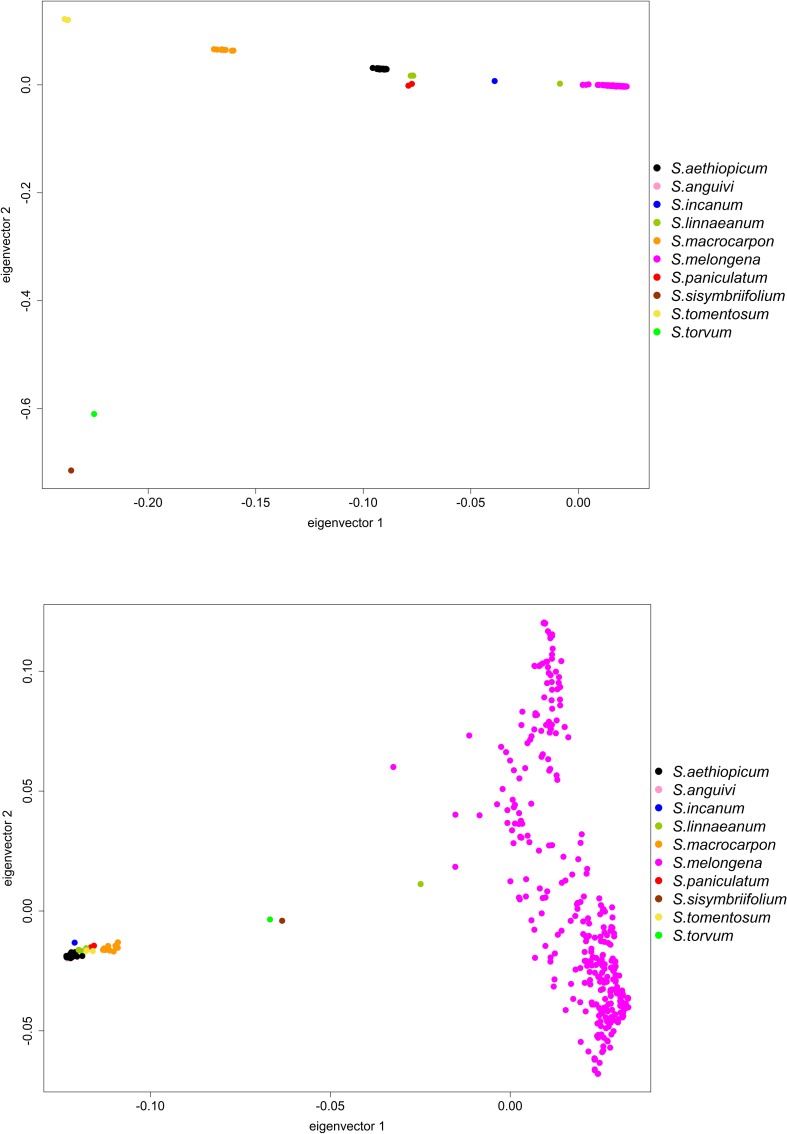
PCA visualization of the genetic relationships among the accessions of eggplant as well as cultivated and wild related species in study, based on the whole (upper figure) or target (lower figure) SNP datasets. Accessions of *Solanum paniculatum* are mislabeled in the germplasm collection, and actually are *Solanum* sp.

The PCA analysis based on the whole eggplant SNPs dataset (Figure 8), largely confirms the grouping of genotypes obtained in the ML-based dendrogram. The first and second principal axes account, respectively, for 13.5 and 11.5% of the genetic variation. In the PCA graph, the two American species *S. sisymbriifolium* and *S. torvum* are clearly separated from the rest of entries. The species of the “Eggplant” clade and “Anguivi” grade are clearly separated by the first component of the PCA, with two exceptions: the *S. linnaeanum* accession GPE003740 as well as the entries of *S. aethiopicum* and *S. anguivi* clustering together. The closest species to *S. melongena* are the other “Eggplant” clade species *S. incanum* and *S. linnaeanum*, as well as the two accessions labeled as *S. paniculatum*, followed by the “Anguivi” grade *S. aethiopicum* and *S. anguivi*, the entries of *S. macrocarpon* and the ones of *S. tomentosum*.

The first and second components of the eggplant PCA based on target SNPs account, respectively, for 29.1 and 11.3% of the genetic variation. As observed with the ML-based dendrogram, the PCA displays considerable differences with the one obtained with the whole SNPs dataset (Figure 8). Indeed, the first component separates *S. melongen*a and, surprisingly, *S. linnaeanum* accession GPE003740 from the other species. *Solanum melongena* is spread over a large area of the PCA graph with a wide range of values for both the components. The rest of species of the “Eggplant” clade and the “Anguivi” grade cluster together and in some cases are overlapped. Among this group of species, the closest to *S. melongena* are *S. macrocarpon*, followed *S. tomentosum*, *S. linnaeanum*, *S. incanum*, and finally *S. aethiopicum* and *S. anguivi*. Surprisingly, the American species *S. sisymbriifolium* and *S. torvum* plot in an intermediate area between *S. melongena* and the rest of Old World species (Figure 8).

## Discussion

The single primer enrichment technology, recently developed by Nugen, has been used up to now for biomedical applications (Scolnick et al., 2015; Nairismägi et al., 2016), and in plants, for genotyping in monocot (*Z. mays*) lines and in a natural black poplar (*P. nigra*) population (Scaglione et al., 2019). Despite its potential interest, no information is available on its performance for characterizing large germplasm sets from crop plants. Our main goal was to assess the reliability and efficiency of the SPET technique for high-throughput genotyping of a large (822) set of accessions of tomato, eggplant and their cultivated and wild relatives. For this purpose, we evaluated the robustness of the technique and the diversity, heterozygosity and genetic relationships within the germplasm included in this study, comparing with the data reported in the literature.

Single primer enrichment technology is a robust method, performing well with DNA samples prepared by different laboratories using different DNA mini-preparation protocols, which is a prerequisite for large multicenter, and collaborative studies on plant genetic resources. Indeed, based on tomato samples extracted with different protocols, both the number of reads and the mapping percentage were relatively stable. Additionally, a very low level of missing data (1.18% for tomato, and 0.02% for eggplant) was observed when genotyping an intra-specific diversity panel, and a still acceptable (<5%) level was observed when genotyping species such as *S. pennellii* or *S. macrocarpon* which show 2.7 and 3.4 million years of divergence from tomato and eggplant, respectively (Kamenetzky et al., 2010; Särkinen et al., 2013).

Single primer enrichment technology combines in a single approach both targeted analysis of SNPs, thus being comparable with genotyping arrays, and complexity reduction typical of GBS approaches (Scheben et al., 2017). Furthermore, SPET provides the ability of multiplexing thousands of samples in a single sequencing run, which can be genotyped with tens of thousands of probes, and with a good coverage at target sites. Finally, thanks to the sequencing of the genomic regions around the target SNPs, SPET allows the discovery of thousands of novel SNPs not originally included in the panel.

Compared to other crop species, both tomato and eggplant are known to have experienced a dramatic reduction of the genetic variability due to anthropogenic selection (Williams and St Clair, 1993; Cericola et al., 2013; Flint-Garcia, 2013), and hence present a lower frequency of SNPs than their wild species. The tomato and eggplant panels designed for this work, which originally targeted 5k SNPs for each species, allowed the discovery of 7,427 and 26,103 additional non-target SNPs, respectively, in the tomato and eggplant sets. Of these, 2,224 and 3,292 were detected only in *S. lycopersicum* and *S. melongena*, respectively, while 7,130 and 24,892 were found to be shared in the remaining species, belonging to the tomato and eggplant genepools, respectively. This indicates that the technique enables the discovery of high numbers of novel polymorphisms, even in gene pools that came across severe bottlenecks during domestication, migration and selection.

Most of the accessions of the largely autogamous *S. lycopersicum* and *S. melongena* (Chen et al., 2007; Daunay and Hazra, 2012; Acquadro et al., 2017) showed a low heterozygosity (on average 0.65 and 0.67%, respectively), in agreement with previous reports (Sim et al., 2012; Vilanova et al., 2012; Aflitos et al., 2014; Acquadro et al., 2017). Only a few accessions of both crops displayed higher values, up to 8.7% for tomato and 7.1% for eggplant, which probably reflect recent events of hybridization either before collection or during germplasm multiplication. Thus, the technique allows the identification of segregating accessions and may guide sampling during seed multiplication. For the tomato germplasm set, the accessions of *S. pimpinellifolium*, the closest wild relative of *S. lycopersicum*, exhibited on average an intermediate (4.7%) level of heterozygosity between *S. lycopersicum* and the other wild species, some of which are self-incompatible (Chen and Tanksley, 2004). In the two additional cultivated species of eggplant, i.e., *S. aethiopicum* and *S. macrocarpon*, a higher heterozygosity was detected, being on average, of 1.9 and 2.5%, respectively. This is attributable to the more limited anthropogenic selection on these species and to their higher rate of allogamy (Daunay et al., 2001). These values are slightly lower than the ones previously reported (Acquadro et al., 2017), based on the RAD-sequencing technique which provides a randomized representation of the genome.

Genetic relationships among the accessions in the study were explored by constructing ML phylogenetic trees and PCA analyses, based on both the whole set or just the target SNPs. The stringent criteria adopted to select the set of SNPs consistently reduced the frequency of missing data. A total of 358 of 361 accessions of *S. lycopersicum* displayed on average 1,18% of missing data and their frequency was up to 3.3% in the closely related species *S. pimpinellifolium.* Higher values were detected in three tomato accessions (GPT141120, GPT130370 and GPT124880) due to the low number of reads obtained following sequencing. However, this did not affect their clustering with the other accessions of *S. lycopersicum* when ML tree and PCA were based on both the whole SNPs dataset or only the target SNPs. Indeed, several studies have explored how missing data may impact phylogenetic analyses using both empirical and simulated data, and suggest that it is possible to include taxa that have large amounts of missing data without ill effects (Wiens and Moens, 2008; Lynch and Wagner, 2010; Thomson and Shaffer, 2010; Wiens and Morrill, 2011). In the remaining accessions, the missing data ranged from 3.0 to 17.7% and reached the highest values in *S. habrochaites*, the most evolutionary divergent wild species among those evaluated (Aflitos et al., 2014; Beddows et al., 2017).

In almost all accessions of *S. melongena*, and the ones of the cultivated *S. aethiopicum*, very low levels (0.02 and 0.7%, respectively) of missing data were detected; in the other cultivated eggplant (*S*. *macrocarpon*) their frequency was on average 4.5%. The missing data varied in entries of the wild species and reached the highest values in the New World native species *S. torvum* (21.2%) and *S*. *sisymbriifolium* (22.7%), characterized by greater evolutionary divergence (Vorontsova et al., 2013; Acquadro et al., 2017). In two accessions of *S. melongena* and one of *S. linnaeanum*, an unexpected high frequency of missing data was observed, which was attributable to the low number of reads obtained. As observed for tomato, the two *S. melongena* accessions always clustered with the ones of the other accessions of this species, while the *S. linnaeanum* accession clustered with the others of the same species when the whole SNP dataset was used, but separately when only target SNPs were used. This suggests that, when a limited number of reads is obtained, the analysis based on the whole set of SNPs is less prone to misclassification of highly diverse genotypes.

Single primer enrichment technology genotyping with the whole set of SNPs or just the target SNPs proved to be a powerful tool for the identification of duplicates and mislabeled accessions. The two replicates of the *S. lycopercicum* accession “Heinz 1706,” as well as the three replicates of the *S. melongena* breeding line “67/3” clustered together, with SNP polymorphism ranging from 0.1 to less than 0.2%. Furthermore, the two accessions initially labeled as *S. paniculatum*, a close relative of *S. torvum* (Miz et al., 2008), did not cluster with the latter nor with the other New World species *S. sisymbriifolium.* Instead, the SPET genotyping data suggested that these two accessions labeled as *S. paniculatum* correspond to an undetermined species (*Solanum* sp.) closely related to the eggplant clade or to the closely related “Anguivi” grade (Vorontsova et al., 2013). Closer passport and phenotypic inspection confirmed the taxonomic mislabeling of these two accessions. Additionally, three *S. melongena* accessions initially mislabeled as *S. aethiopicum* in the germplasm bank listing were correctly clustered with the rest of *S. melongena* accessions, demonstrating that SPET is also a powerful technique (Mason et al., 2015) for detecting misclassified accessions for which no or few characterization data are available.

### Tomato and Eggplant Diversity

Our results of the tomato and eggplant genotyping with the SPET platform are largely congruent with previous results on the knowledge of diversity in these groups (Lester and Thitai, 1989; Peralta et al., 2008; Rodriguez et al., 2009; Aflitos et al., 2014; Dodsworth et al., 2016; Acquadro et al., 2017; Gramazio et al., 2017). On the basis of both ML dendrogram and PCA analysis with all SNPs, the *S. lycopersicum* accessions clustered separately from all the other species and grouped in a single branch, revealing a low diversity. *S. pimpinellifolium*, native to Peru and Ecuador (Grandillo et al., 2011), is the only red-fruited wild species and it is the nearest wild relative to the cultivated tomato. In agreement with previous findings (Rodriguez et al., 2009; Aflitos et al., 2014; Dodsworth et al., 2016), its accessions were found to cluster close to *S. lycopersicum* in the ML dendrogram. It has been reported that the *S. pimpinellifolium* accessions are divided in three main genetic groups, corresponding to the environmental differences found in the coastal regions of Northern Ecuador, in the mountain region of Southern Ecuador and Northern Peru, and the coastal region of Peru (Blanca et al., 2015). Due to the smaller number of accessions of this species under study, we were not able to identify these three groups, however, the ML dendrogram detected two *S. pimpinellifolium* sub-clusters which partially correlate with the Ecuadorian and Peruvian origin of the entries. On the basis of PCA analysis, some *S. pimpinellifolium* and *S. lycopersicum* accessions were intermingled. This is in agreement with previous findings that the genome of the two species shows only 0.6% nucleotide divergence and signs of recent admixture (The Tomato Genome Consortium, 2012). Furthermore, due to the absence of crossing barriers between the two species, introgression events from *S. pimpinellifolium* into *S. lycopersicum* have probably occurred throughout the history of tomato domestication (The Tomato Genome Consortium, 2012).

The position of the rest of wild species in the dendrogram and PCA analyses with all SNPs is in agreement with previous studies (Rodriguez et al., 2009; Aflitos et al., 2014; Dodsworth et al., 2016; Beddows et al., 2017), i.e., “Arcanum” group species were found to be closer to the “Lycopersicon” group than the “Eriopersicon” and “Neolycopersicon”, and the two sister species *S. chmielewskii* and *S. neorickii* clustered together (Dodsworth et al., 2016).

*Solanum melongena* accessions of both Oriental and European origin, producing fruits of different shape and color, clustered separately from all the other cultivated and wild species in both the ML dendrogram and PCA obtained by using the whole SNP dataset. The remaining species fell in the dendrogram and PCA plots according to their expected positions (Acquadro et al., 2017; Gramazio et al., 2017). Among the eggplant relatives, our data showed that *S. incanum* was the closest species to eggplant, followed by the other “Eggplant” clade species (S. *linnaeanum*), as reported by other studies (Lester and Hasan, 1991; Weese and Bohs, 2010).

Contrasting results (Sakata et al., 1991; Sakata and Lester, 1997; Isshiki et al., 2008; Meyer et al., 2012; Vorontsova et al., 2013) have been reported on the relationships between *S. melongena* and the other two cultivated eggplants: *S. aethiopicum* (scarlet eggplant) and *S*. *macrocarpon* (gboma eggplant). In recent studies, it has been reported that the latter is genetically closer to eggplant (Acquadro et al., 2017). However, by applying SSR or SNP genotyping, different results were obtained (Gramazio et al., 2017). Our SPET-based clustering highlighted that the three cultivated species belong to clearly separate groups that probably diverged at similar times. As expected (Lester and Niakan, 1986; Lester and Hasan, 1991; Syfert et al., 2016), the accessions of *S. aethiopicum* resulted intermingled with those of its wild ancestor *S. anguivi*, indicating a probable genetic flux between the two species (Plazas et al., 2014). Also, *S. tomentosum* clustered in the “Anguivi” grade, confirming previous results (Vorontsova et al., 2013; Acquadro et al., 2017). As previously reported (Acquadro et al., 2017), the wild *S*. *sisymbriifolium* and *S*. *torvum*, native of South and Central America and representing sources of resistance to several diseases (Daunay and Hazra, 2012) formed a separate group following both the ML tree and PCA analyses.

In both the tomato and eggplant genepools, the ML dendrograms and PCA analyses performed on total SNP datasets provided a clustering similar to the one obtained on the basis of the target SNPs. However, some differences were evident: target SNPs provided a higher intra-specific discrimination within tomato and eggplant, accompanied by a less clear clustering of the other cultivated and wild species. The higher discrimination of different eggplant accession was particularly evident in the PCA analysis (Figure 8), and their genetic differentiation was maximized in the ML dendrogram as well (Figure 7). On the other hand, the use of target SNPs decreased the discrimination between the cultivated *S. aethiopicum* and *S. macrocarpon*, and the wild species belonging to *S. melongena* genepool clustered into a single branch of the ML tree, showing a strong clustering in the PCA graph.

The differences observed when using the target vs. whole SNP datasets are attributable to the number and the genetic distance of the resequenced genotypes used for the initial identification of the polymorphic sites for SPET set up. In tomato, the SPET panel was based on a majority of resequencing data from *S. lycopersicum* (including var. *cerasiforme*) and some *S. pimpinellifolium*, while the eggplant panel was based on resequencing of eight *S. melongena* and one *S. incanum* accessions. Thus, in *S. lycopersicum* a clear assessment of the genetic relationships among species of the tomato genepool was obtained when SPET was based on both the whole dataset and target SNPs, although the genetic differentiation within tomato accessions was to a certain extent increased by the use of target SNPs. Vice versa, in eggplant, the whole SNP dataset was more informative for phylogenetic studies, while the use of target SNPs allowed a huge increase in the detection of genetic variation within accessions of *S. melongena*, as required for GWA studies.

## Conclusion

Recently, a targeted genotyping approach (SPET) was released, but very limited information of its performance for high-throughput genotyping in plants is presently available. We assessed the efficiency and robustness of this technique in analyzing the genetic diversity in hundreds of *S. lycopersicum* and *S. melongena* germplasm accessions and of their cultivated and wild relatives maintained in genebanks. A high number of both target and non-target polymorphisms were analyzed by ML dendrograms and PCA analyses and the two approaches were complementary in the interpretation of data.

Our results demonstrate that SPET represents a valid alternative to random complexity reduction methods and arrays, and it allows users to customize the panel of target markers and provides reliable fingerprinting of accessions maintained in genebanks. Our results also demonstrate the transferability of the panels to closely related species, and that the number and genetic distances of the resequenced genotypes used for identifying the target SNPs play a significant role. We designed panels enriched in intra-specific SNPs reasoning that additional inter-specific ones would be more likely to be discovered in the surrounding sequenced regions, which was indeed the case. The use of the whole SNP dataset is more appropriate for broad phylogenetic studies, while the use of target SNPs, boosting the intra-specific discrimination, for domestication and genome-wide association studies. The SPET technology made it also possible to clearly separate the different species, confirmed established phylogenetic relationships among different species and taxonomic groups, resolved the mislabeling of entries, and demonstrated its high reproducibility when applied on replicates of the same accession, proving its usefulness for the high-throughput genotyping, management, and enhancement of genebank collections.

## Data Availability

The datasets generated for this study can be found in the BioProject ID: PRJNA542237 for tomato data and BioProject ID: PRJNA542231 for eggplant data.

## Author Contributions

GG, SL, JP, and GLR conceived and designed the research. LT, LaB, GLR, DA, PG, SV, MD, OD, PM, and PF provided the plant material and extracted the DNA samples. DS performed the SPET panel design. LoB, GA, EP, and AA analyzed the data. LoB, GG, JP, and SL wrote the manuscript. PG, SV, AA, EP, MD, GLR, and LT reviewed and edited the manuscript. All authors read and approved the manuscript.

## Conflict of Interest Statement

DS was employed by the company IGA Technology Services Srl. The remaining authors declare that the research was conducted in the absence of any commercial or financial relationships that could be construed as a potential conflict of interest.

## Footnotes

1 http://solgenomics.net

2 ftp://ftp.solgenomics.net/genomes/

3 http://www.g2p-sol.eu/

4 https://software.broadinstitute.org/gatk/best-practices/workflow?id=11145

## Supplementary Material

TABLE S1 All the resequenced accessions of tomato used for SNPs mining.

TABLE S2 Tomato genepool accessions and SNPs metrics.

TABLE S3 Eggplant genepool accessions and SNPs metrics.

TABLE S4 Robustness of the final tomato SPET assay using DNAs prepared with different methods and by different laboratories.

TABLE S5 PIC values of the whole SNPs identified in the tomato genepool.

TABLE S6 PIC values of the target SNPs identified in the tomato genepool.

TABLE S7 PIC values of the whole SNPs identified in the eggplant genepool.

TABLE S8 PIC values of the target SNPs identified in the eggplant genepool.

FILE S1 Sequencing yields, coverage analysis and filtering for probe selection in tomato and eggplant.

FILE S2 Phylip formatted input files used for generating phylogenetic tree by maximum likelihood (ML).

FILE S3 Sequences of tomato and eggplant probes used for SPET assay.
